# Association Between Triglyceride–Glucose Related Index and Endometriosis Varies According to Educational Level

**DOI:** 10.3390/nu17040670

**Published:** 2025-02-13

**Authors:** Chuan Lin, Qian Wu, Zhao Luo, Jiacheng Du, Seong-Tshool Hong, Hee-Suk Chae

**Affiliations:** 1 Department of Biomedical Sciences, Institute for Medical Science, Jeonbuk National University Medical School, Jeonju 501-757, Republic of Korea; mumuli415@163.com; 2 Research Institute of Clinical Medicine, Department of Orthopedics, Jeonbuk National University Medical School, Jeonju 501-757, Republic of Korea; 3 Department of Urology, Jeonbuk National University Medical School, Jeonju 501-757, Republic of Korea; 4 Research Institute of Clinical Medicine, Jeonbuk National University Medical School, Jeonju 501-757, Republic of Korea; 5 Department of Obstetrics and Gynecology, Biomedical Research Institute, Jeonbuk National University, Jeonju 501-757, Republic of Korea

**Keywords:** endometriosis, TyG, TyG-WHtR, TyG-WC, TyG-BMI, high educational level, NHANES

## Abstract

Background: The association between the triglyceride-glucose (TyG) level, its obesity-related derivatives, and the occurrence of endometriosis (EMS) remains ambiguous, particularly in individuals with higher levels of education. This study sought to explore the relationship between TyG, its obesity-related derivatives, and EMS across various educational backgrounds. Methods: This study utilized a substantial dataset obtained from four cycles of the National Health and Nutrition Examination Survey (NHANES) conducted between 1999 and 2006. To explore the relationship between TyG, its obesity-related derivatives, and EMS, we employed a variety of analytical methods, including multivariable logistic regression models, smooth curve fitting, threshold effect analysis, and subgroup analysis, which were applied to participants with varying educational levels. Results: Among the 2347 participants, 203 (8.65%) were diagnosed with EMS. In the overall population, only the TyG, TyG-waist-to-height ratio (TyG-WHtR), and TyG-waist circumference (TyG-WC) variables demonstrated a positive association with EMS. However, within the group with high educational attainment, TyG, TyG-WHtR, TyG-WC, and TyG-body mass index (TyG-BMI) all exhibited positive correlations with EMS. These associations remained robust after adjustment for multiple potential confounding variables. The subgroup analysis demonstrated that these associations were consistent across different subgroups (*p* > 0.05). Furthermore, both linear and nonlinear relationships were observed between TyG and its obesity-related derivatives and EMS, as evidenced by the smooth curve fittings and threshold effect analyses. In contrast, no significant associations were identified in the group with lower levels of education. Conclusions: Our study suggests that there is variation in the association between TyG and its obesity-related derivatives and EMS across different educational levels, warranting further investigation. In individuals with higher education, elevated levels of TyG and its obesity-related derivatives were associated with a higher prevalence of EMS. Conversely, this correlation was not observed among those with lower educational levels.

## 1. Introduction

Endometriosis (EMS) is a chronic illness with an unclear etiology, marked by functional endometrial tissue located outside the uterine cavity. This abnormal tissue can provoke local inflammation, adhesions, fibrosis, scar formation, neuronal infiltration, and anatomical distortion, leading to pain and infertility [1,2]. The worldwide prevalence of endometriosis is estimated to be about 5–10%, affecting approximately 190 million women of reproductive age [3]. Studies indicate that the annual healthcare expenses for endometriosis patients amount to an average of approximately EUR 3113, with the United States facing a total annual socioeconomic burden that is as high as EUR 49.6 billion [4]. The clinical presentation of EMS is highly variable, with the early stages often manifesting as non-specific pelvic pain. Furthermore, there is a lack of specific biomarkers for the disease. The gold standard for diagnosis remains the direct visualization of lesions through laparoscopy, with confirmation of the diagnosis via histopathology [5]. Nevertheless, it has been documented that this diagnostic approach can result in a delay of approximately 7–8 years from the initial onset of symptoms to a confirmed diagnosis, which impedes early detection of the disease [6]. In light of these considerations, international guidelines now recommend a non-surgical approach to diagnosis based on the analysis of symptoms, a physical examination, and imaging results. This approach is intended to facilitate early diagnosis, prompt intervention, and ultimately slow the disease’s progression, thereby improving the quality of life of patients [7]. Given these challenges, researching the factors associated with EMS is crucial. Identifying these factors could aid in recognizing high-risk populations, facilitating early diagnosis and intervention, formulating effective prevention strategies, reducing the incidence of the disease, and alleviating the associated socioeconomic burden.

The pathogenesis of EMS likely involves multiple pathways, including inflammation, the immune response, genetics, endocrine factors, and environmental influences. Among these, inflammation plays a central role in EMS. During the formation of ectopic lesions, the excessive recruitment of inflammatory cells occurs in the affected area, where they secrete inflammatory cytokines. Neutrophils release IL-8 and IL-17α, contributing to the persistence of the inflammation. These cytokines in turn exacerbate the pathological processes in ectopic lesions, such as lesion growth and implantation. In the inflammatory response, endothelial cells express adhesion molecules and secrete IL-6, facilitating the migration of inflammatory cells. This helps maintain a local, chronic inflammatory microenvironment, exacerbating the invasion of normal tissue by ectopic lesions and increasing the extent of systemic chronic low-grade inflammation. Recent research suggests that glucose and lipid metabolism disorders may lead to oxidative stress and endothelial dysfunction, triggering inflammatory signaling pathways [8]. This results in the excessive release of pro-inflammatory cytokines, such as TNF-α, inducing chronic low-grade inflammation and oxidative stress [9], reducing the insulin sensitivity in EMS cells, and ultimately promoting the development of EMS [10]. Triglyceride-glucose (TyG), which reflects the triglyceride (TG) and fasting glucose (FG) levels in the body, was initially proposed by Simental-Mendia et al. as a means of identifying insulin resistance [11]. TyG has gained attention in the field of insulin resistance assessment due to its simplicity and effectiveness [12]. When combined with obesity-related indicators such as the waist-to-height ratio (WHtR), waist circumference (WC), and body mass index (BMI), the derived TyG index has demonstrated excellent performance in identifying insulin resistance [13] as well as in predicting outcomes related to cardiovascular disease [14], stroke [15], arthritis [16], and metabolic dysfunction-associated fatty liver disease [17]. However, it is important to note that the association between TyG and various diseases may not be consistent across different populations. For instance, Liu et al. discovered that TyG’s obesity-related derivatives were markedly linked to depression in premenopausal women, but this correlation was not statistically significant in postmenopausal women [18]. Similarly, Yao’s research indicated that the relationship between TyG and mortality in US patients with type 2 diabetes varies with age. A higher TyG level was associated with increased mortality in individuals under 65 years of age, whereas this association was not confirmed in those aged 65 years and above [19].

Although previous studies have preliminarily explored the association between TyG and EMS [20,21], the relationship between TyG-derived obesity-related indices (such as TyG-WC, TyG-WHtR, and TyG-BMI) and EMS has not been thoroughly investigated. These obesity-related indices, as comprehensive indicators reflecting an individual’s metabolic status and body fat distribution, may provide deeper insights into the potential link between metabolic abnormalities and EMS. Thus, by addressing these indices, our study fills an important research gap. Furthermore, a patient’s educational level, as a critical socioeconomic factor, is closely associated with their health outcomes. However, few studies have explored the potential moderating role of the educational level in the relationship between TyG, its obesity-related derivatives, and EMS. To address these gaps, we utilized data from the National Health and Nutrition Examination Survey (NHANES) to analyze the association between TyG, its obesity-related derivatives, and EMS, with a focus on how this association differs across various levels of educational attainment. This research aimed to offer new insights and strategies for the prevention and treatment of EMS.

## 2. Methods

### 2.1. Data Source

The data were sourced from the NHANES, a nationwide epidemiological survey on health and nutrition conducted by the National Center for Health Statistics. This survey aims to collect health data and related lifestyle information from the US population, with updates every two years. The NHANES includes a broad spectrum of non-institutionalized individuals of all ages living in the US, with particular attention paid to minority groups, low-income individuals, and the elderly to ensure adequate representation. The survey collects detailed demographic information, health status data, physical examination findings, and laboratory test results. All data are recorded in real time into a computerized system and subjected to rigorous quality control processes. After anonymization, these data are made available to public health researchers and policymakers. This survey received approval from the Research Ethics Review Board of the National Center for Health Statistics (https://www.cdc.gov/nchs/nhanes/index.html).

### 2.2. Study Participants

**Figure 1:**
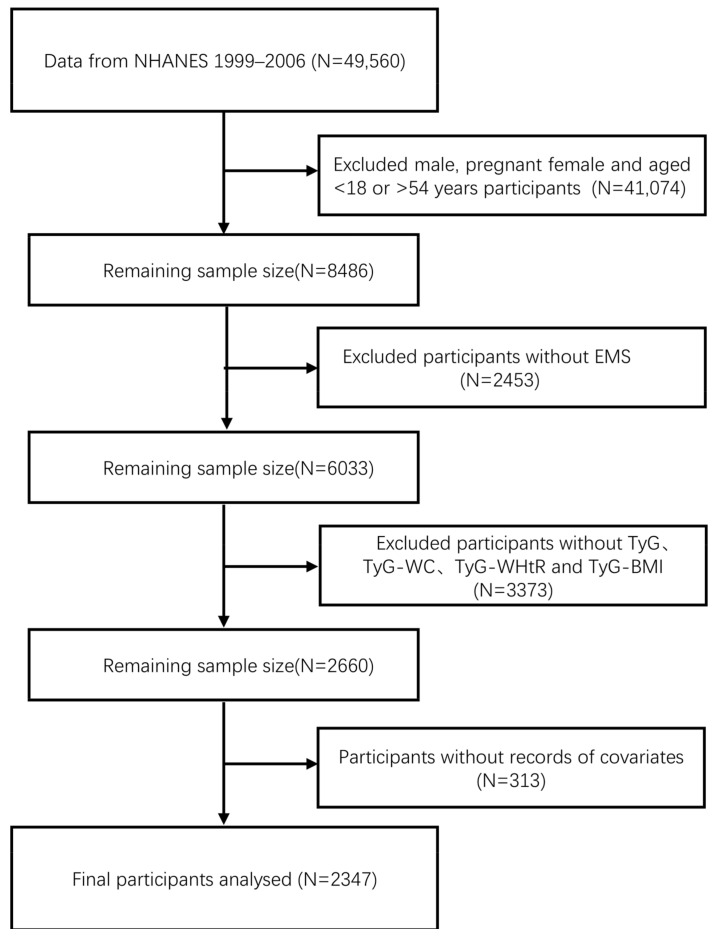
Flowchart of participant selection from NHANES (1999–2006).

This study analyzed data from four consecutive NHANES cycles conducted between 1999 and 2006. Initially, 49,560 individuals were included in the sample. Since the objective was to investigate the relationship between TyG and its obesity-related derivatives and EMS, we excluded the following groups: (1) all male participants, females younger than 18 or older than 54, and pregnant women; (2) individuals without EMS diagnosis data; (3) participants missing essential information required to calculate TyG and its derivatives, such as TGs, FG, WC, height, and BMI; and (4) those missing data on their educational levels and other covariates. Ultimately, this study included a total of 2347 participants (Figure 1).

### 2.3. Measurement of TyG and Its Obesity-Related Derivatives

TyG is an indicator which is calculated based on the levels of fasting triglycerides (TGs) and fasting glucose (FG). Its combination with obesity indicators, namely the waist-to-height ratio (WHtR), WC, and body mass index (BMI), was considered in order to obtain a composite indicator of TyG and obesity. We refer to TyG-WHtR, TyG-WC, and TyG-BMI collectively as TyG obesity-related derivatives [22]. The participants were asked to fast for 8–12 h while their TGs and FG were measured. Blood samples were drawn by trained personnel at mobile examination centers through venipuncture. The samples were then immediately frozen and stored until they were transported to designated laboratories for analysis. The TG concentrations in the blood samples were measured using an enzymatic method with an automated biochemical analyzer, and the results were recorded in mg/dL and stored in a TRIGLY file. The FG levels were measured using the hexokinase method, and they were also reported in mg/dL and archived in a GLU file. Additionally, the WC, height, and weight were measured following standardized protocols, with the results noted in centimeters (cm), meters (m), and kilograms (kg), respectively. The WHtR was calculated as the ratio of WC to height, while the BMI was determined by dividing the weight by the height squared. The final formulas used to calculate TyG and its obesity-related derivatives are shown below [23,24]:

$$\mathrm{TyG}=\ln\left(\mathrm{TG}\;\times \frac{\mathrm{FG}}{2}\right)$$

TyG-WHtR=TyG×WHtR

TyG-WC=TyG×WC

TyG-BMI=TyG×BMI

Given the large values for TyG-BMI and TyG-WC, a natural logarithm (Ln) transformation was applied, which did not alter the original data’s trends [18,25].

### 2.4. Definitions of EMS, Low Educational Level, and High Educational Level

The diagnosis of EMS and the evaluation of the educational levels were also carried out at mobile examination centers. The diagnosis of endometriosis was based on self-reporting from questionnaires in the NHANES database. Specifically, when asked “Have you ever been told by a doctor that you have endometriosis?” participants who answered “yes” were classified as having endometriosis [26], and the relevant data were stored in the RHQ file. The demographic questionnaire detailed the participants’ educational information, which was categorized based on the level of education: less than 9th grade; 9–11th grade; high school graduate, GED, or equivalent; some college or an associate degree; and college graduate or higher. Following the International Standard Classification of Education 3 guidelines, participants with fewer than 12 years of education were classified as having a low educational level (LEL), while those with more than 12 years of education were classified as having a high educational level (HEL) [27].

### 2.5. Covariates

The adjusted covariates included demographic information, namely age, race, educational level, marital status, and poverty-income ratio (PIR); lifestyle factors (smoking, alcohol intake, vigorous activity, and moderate activity); and health status (diabetes, hypertension, age at menarche, and history of pregnancy). The PIR is the ratio of the annual household income to the federal poverty line for the corresponding household size, reflecting the economic status of a household or individual, and it was categorized into three groups: 0–1.29, 1.3–3.49, and >3.5. Variables such as alcohol consumption, smoking, physical activity, and hypertension were defined with reference to a range of studies using NHANES data. Alcohol consumption was defined as having consumed at least 12 drinks in one’s lifetime [28], where one drink was equivalent to 12 ounces of beer, 4 ounces of wine, or 1 ounce of liquor. Relevant data were obtained from files labeled “ALQ”. Smoking was defined as having smoked at least 100 cigarettes in one’s lifetime [29]. Physical activity was categorized into vigorous and moderate activity [30]. Vigorous activity was defined as exercises such as cycling or swimming performed in the past 30 days which significantly increased an individual’s heart rate or respiratory frequency and were performed for more than 10 min per session. Moderate activity referred to exercises such as golfing or brisk walking performed in the past 30 days which moderately increased a participant’s heart rate or respiratory frequency. Hypertension was determined based on the responses to the questionnaire [31,32]. Participants who answered “yes” to the question “Have you ever been told by a doctor that you have hypertension?” were classified as hypertensive; otherwise, they were classified as non-hypertensive. Similarly, diabetes was identified through self-reporting, where the participants were asked, “Has a doctor ever informed you that you have hypertension or diabetes?” A “yes” response indicated a diagnosis of hypertension or diabetes.

### 2.6. Statistical Methods

Normality and homogeneity of variance tests were performed on continuous variables. For normally distributed continuous variables, we employed the mean and standard deviation to describe the data and utilized a *t*-test for inter-group comparisons. For non-normally distributed variables, we used the median and interquartile range (IQR) to describe the data and employed the Mann–Whitney U test. Multivariable logistic regression models were used to analyze the odds ratios (ORs) and 95% confidence intervals (CIs) of the associations between TyG, its obesity-related derivatives, and EMS at different educational levels. Three models were developed. Model 1 was used as an unadjusted model to establish baseline associations without considering other variables. Model 2 included the demographic variables (age, race, marital status, and family PIR) to account for their potential effects on the main associations. Model 3 further incorporated the lifestyle factors (smoking, alcohol consumption, and physical activity) and health conditions (including BMI, diabetes, and hypertension). This stepwise modeling approach is widely used in observational studies to facilitate an understanding of the relationships being studied [33,34]. TyG and its obesity-related derivatives were grouped into quartiles (Q1, Q2, Q3, and Q4), with Q1 serving as the reference group for trend tests. Subgroup analyses were carried out based on age, race, marital status, PIR, alcohol consumption, smoking, hypertension, and diabetes to explore potential effect modifications. The associations between TyG, its obesity-related derivatives, and EMS were visualized using generalized additive models. Threshold effect analyses and log-likelihood ratio tests were used to explore potential nonlinear relationships between TyG, its obesity-related derivatives, and EMS. The statistical analyses were carried out using R software (version 4.1.1) and EmpowerStats (version 4.2). A two-tailed *p* value < 0.05 was deemed statistically significant.

## 3. Results

### 3.1. Baseline Features of Participants

This study included 2347 participants, with 203 who were diagnosed with EMS, accounting for 8.65% of the study population. Moreover, 1351 had an educational level beyond high school. The participants had an average age of 37.00 (28.00–45.00) years, with non-Hispanic white individuals comprising the largest demographic group (47.64%). The HEL group had a higher percentage of individuals who consumed alcohol (28.29% versus 19.69%, *p* = 0.004), but lower proportions in terms of smoking (34.79% versus 43.98%, *p* < 0.001), the prevalence of hypertension (18.65% versus 21.89%, *p* < 0.001), the prevalence of diabetes (3.70% versus 6.22%), and previous pregnancies (76.61% versus 92.57%) compared with the LEL group. Additionally, the HEL group had lower values for the BMI, WC, FG, TyG, and its obesity-related derivatives compared with the LEL group (*p* < 0.001) (Table 1).

### 3.2. Relationship Between TyG and Its Obesity-Related Derivatives and EMS

To explore the relationship between TyG and its obesity-related derivatives and EMS, we established three different models and performed a logistic regression analysis after adjusting for relevant covariates. As shown in Table 2, in the overall population, EMS was positively correlated with TyG, TyG-WHtR, and LnTyG-WC (*p* < 0.05). In the three mod-els for the HEL group, TyG, LnTyG-WC, TyG-WHtR, and LnTyG-BMI all showed associations with EMS. Furthermore, when TyG and its obesity-related derivatives were divided into quartiles, a positive association with EMS was observed in the Q4 group for each of these indices, and all showed an increasing trend (*p* for trend < 0.05). This indicates that after adjustment for the variables, each unit increase in TyG, TyG-WHtR, LnTyG-WC, and LnTyG-BMI in the Q4 group compared with the Q1 group would correspondingly increase the odds of developing EMS by 172%, 89%, 138%, and 154%, respectively. However, there was no significant correlation between TyG, its obesity-related derivatives, and EMS within the LEL group. Furthermore, a univariate logistic regression analysis was performed for various populations to identify variables which were significantly associated with EMS (*p* < 0.05) (Table S1). The variables which were found to be significantly associated in the univariate analysis were subsequently included in the multivariate logistic regression analysis to further evaluate the association between the TyG index and its derivatives and EMS (Table S2).

### 3.3. Subgroup Analysis

**Figure 2:**
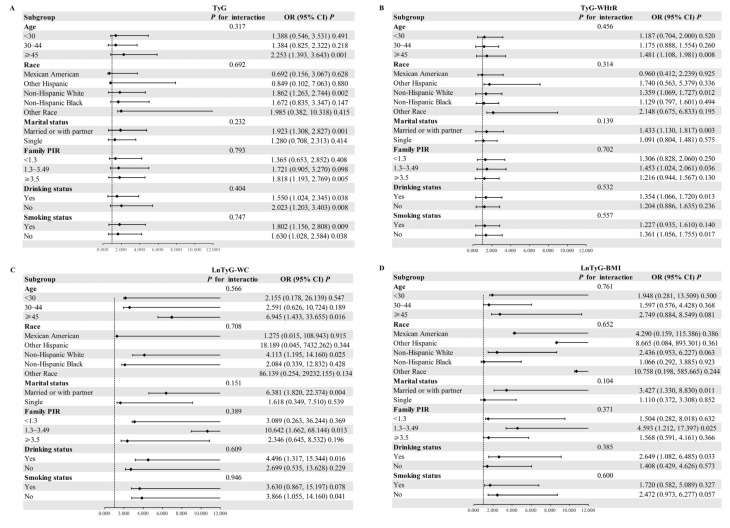
Forest plot of subgroup analysis for relationships between TyG and its obesity-related derivatives and EMS in HEL group. (**A**) Forest plot of subgroup analysis for relationships between TyG and EMS; (**B**) Forest plot of subgroup analysis for relationships between TyG-WHtR and EMS; (**C**) Forest plot of subgroup analysis for relationships between LnTyG-WC and EMS; (**D**) Forest plot of subgroup analysis for relationships between LnTyG-BMI and EMS.

In the HEL population, a subgroup analysis was conducted to evaluate the association between TyG and its obesity-related derivatives and EMS (Figure 2). Age, race, marital status, income level, smoking status, and drinking habits were selected as stratification variables, and the participants were assigned to different subgroups. The analysis was adjusted for other covariates aside from the stratification variables. The results showed that the positive association between TyG and its obesity-related derivates and EMS remained stable across the different demographic and health characteristics. Age, race, marital status, income level, smoking, and drinking did not significantly modify this association (*p* for interaction > 0.05).

### 3.4. Smooth Curve Fitting and Threshold Effect Analysis

**Figure 3:**
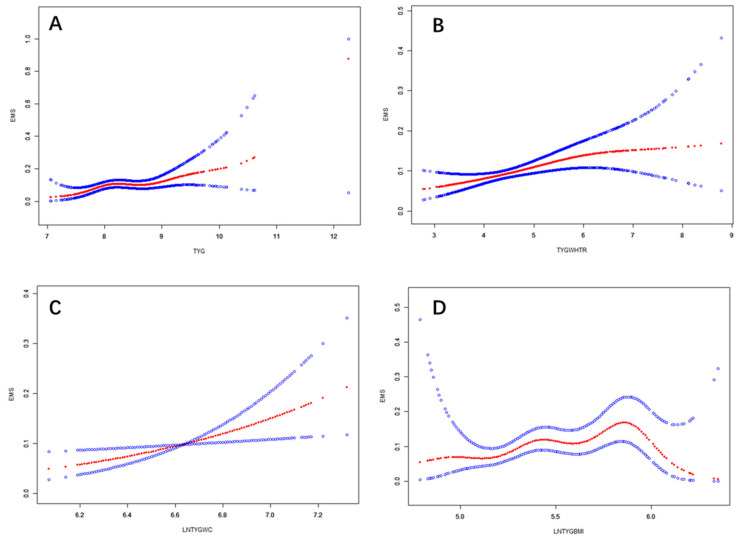
Smooth curve fitting for TyG and its obesity-related derivatives in HEL group. The blue lines represent the 95% CI, and the red lines represent the smooth curve fits between the variables: (**A**) TyG and EMS; (**B**) TyG-WHtR and EMS; (**C**) LnTyG-WC and EMS; and (**D**) LnTyG-BMI and EMS.

After adjusting for covariates in the HEL group, we established smooth curve models and conducted threshold effect analyses to visually represent the correlations between TyG and its obesity-related derivatives and EMS (Figure 3). The results revealed that the log-likelihood ratio test *p* values for TyG and LnTyG-WC were 0.051 and 0.119, respectively (Table 3), suggesting that a linear effect model should be used to confirm the positive correlation between TyG, LnTyG-WC, and EMS. However, the relationships between TyG-WHtR, LnTyG-BMI, and EMS were nonlinear (log-likelihood ratio test *p* values were 0.041 and 0.009, respectively).

## 4. Discussion

In this research, we investigated the connection between TyG and its obesity-related derivatives and the prevalence of EMS across women with varying educational backgrounds. The findings of this nationwide cross-sectional analysis revealed that particularly among women with higher educational levels, TyG, TyG-WHtR, LnTyG-WC, and LnTyG-BMI were all positively correlated with EMS. Notably, this association remained even after adjusting for relevant variables. Moreover, the subgroup analyses further validated the consistency of this relationship across different subsets of the population. However, no significant connection was identified in women with lower educational attainment. These results imply that there is variation in the association between TyG and its obesity-related derivatives and EMS across different educational levels.

EMS is a complex chronic disease, potentially involving multifactorial interactions among immune, genetic, endocrine, and environmental factors. The lack of specific diagnostic biomarkers and the diverse clinical manifestations make its early diagnosis particularly challenging [35]. Recent studies have utilized the NHANES database to explore the factors associated with EMS, uncovering correlations in areas such as inflammation and metabolism. For instance, Hu et al. found a relationship between the Dietary Inflammatory Index and EMS, suggesting that dietary interventions might improve or alleviate the symptoms of EMS [36]. Li et al. revealed a connection between EMS and metabolic syndrome, highlighting a link between EMS and high TG and suggesting that attention should be paid to the metabolic status of EMS patients [37]. Xu et al. observed an association between EMS and arthritis. Through linkage disequilibrium scoring and Mendelian randomization analysis, they explored the intrinsic genetic relationship between the two diseases. In their study, the link between EMS and arthritis did not seem to be driven by inherent genetics, implying that external factors, particularly inflammation, might play a crucial role in the development of EMS [38].

TyG is a well-researched biomarker of insulin resistance widely recognized for its significant link to various metabolic-related diseases, including cardiovascular conditions and diabetes. Extensive studies have shown that higher levels of TyG are associated with a greater risk of these diseases [39,40]. Combining TyG with obesity indices helps to further refine fat distribution assessment in order to evaluate the correlation between obesity and diseases, proven to be more reliable than the use of TyG alone [41]. A cross-sectional study showed that higher levels of TyG and its obesity-related derivatives are associated with hypertension and cardiovascular diseases, with TyG-WC being most effective in diagnosing hypertension and TyG-WHtR being most effective in diagnosing cardiovascular diseases. Thus, they could aid in the primary prevention of cardiovascular diseases [42]. Another six-year prospective cohort study indicated a positive connection between TyG and its obesity-related derivatives and hypertension, identifying TyG-WHtR as the most valuable predictor of hypertension [43]. Dang et al. reported on the predictive ability of TyG and its related indices for cardiovascular disease mortality, noting that TyG-WC and TyG-WHtR improved the diagnostic efficacy for cardiovascular disease mortality compared with TyG alone, enhancing risk stratification [22]. To date, research on TyG and its derived indices has primarily focused on metabolic-related fields, with limited studies on their relationships with EMS. This study explored the association between TyG and its obesity-related derivatives and the prevalence of EMS in women with different educational levels, revealing significant correlations, particularly in the HEL group. In this group, TyG, TyG-WHtR, LnTyG-WC, and LnTyG-BMI were positively correlated with EMS. This study addresses a gap in the current research, offering new insights for early EMS diagnosis and establishing a basis for exploration of the role of insulin resistance in the pathogenesis of EMS.

The mechanisms underlying the interaction between TyG and its obesity-related derivatives and EMS in highly educated populations remain unclear. Insulin resistance, caused by abnormal glucose and lipid metabolism, could be a key factor in the pathophysiology of EMS [44]. High levels of TyG and its obesity-related derivatives may increase insulin resistance and metabolic syndrome through multiple mechanisms, thereby increasing the risk of developing EMS. Insulin resistance is often accompanied by the release of more pro-inflammatory cytokines from adipocytes [45,46], such as tumor necrosis factor-α, C-reactive protein, and interleukin-6 [47], which are commonly found in EMS patients [9]. This inflammatory environment contributes to the growth and maintenance of ectopic endometrial tissue, exacerbating EMS’s symptoms and progression [48]. Additionally, insulin resistance leads to alterations in the levels of various hormones, including estrogen, testosterone, and insulin-like growth factor. High insulin levels stimulate the ovaries to produce more androgens and inhibit the production of sex hormone-binding globulin [49], thus increasing the levels of free estrogen and testosterone [9]. Elevated estrogen levels upregulate phosphorylated FOXO1 in ectopic endometrial stromal cells (ESCs), promoting the high expression of Rab27b and the secretion of matrix metalloproteinase-9 (MMP9), increasing ESCs’ invasiveness and thus increasing the risk of EMS [50]. These studies provide preliminary insights into the potential mechanisms linking TyG and EMS, but the role of the educational level in this process remains underexplored. Liang et al. identified notable variations in the relationship between SII and EMS when considering different levels of education [51]. Similarly, our study revealed notable differences in the correlation between TyG and its obesity-related derivatives and EMS in women with different educational levels. In the HEL group, the values of TyG, TyG-WHtR, TyG-WC, and TyG-BMI were all lower compared with those in the LEL group. However, a significant positive correlation between high TyG levels and the prevalence of EMS was only found in the HEL group, while this association was absent in the LEL group. The TyG index is considered to reflect abnormalities in glucose and lipid metabolism, which are closely related to chronic inflammation and endocrine disorders. These mechanisms may play a crucial role in the pathophysiology of EMS. However, metabolic and inflammatory responses may be influenced by various factors across populations with different educational levels. For instance, women with higher educational attainment may exhibit distinct patterns of hormonal exposure and stress responses, which could further increase the risk of EMS by affecting inflammatory responses and endocrine function. Higher educational attainment may also be associated with specific environmental exposures, such as occupational stress and prolonged mental burdens, which may interfere with metabolic and inflammatory networks through multiple pathways, thereby amplifying the association between the TyG index and EMS. Additionally, women with higher educational levels generally have greater health awareness, pay greater attention to their health, and are more likely to proactively seek medical care. This proactivity may lead to the earlier detection and diagnosis of EMS, potentially contributing to the observed positive association between TyG-related indices and EMS in the HEL group. Furthermore, differences in dietary habits, lifestyle factors, and socioeconomic status among individuals with varying levels of education may indirectly influence the relationship between the TyG index and EMS (Figure S1).

This study utilized nationally representative population data to explore the association between TyG and its obesity-related derivatives and EMS among women with different educational levels, thereby extending the application of TyG-related indicators in metabolic and disease research. Our findings not only reveal the potential link between TyG indices and EMS but also underscore the critical role of educational attainment as a socioeconomic factor in regulating metabolic health. Among women with higher educational levels, the positive correlation between TyG and its related derivatives and EMS was more pronounced, suggesting that educational attainment may influence disease development through complex metabolic and inflammatory networks. These findings highlight the importance of considering individuals’ educational backgrounds when formulating intervention strategies for metabolic disorders and adopting stratified management approaches. Furthermore, they provide valuable insights for future research and the development of public health policies. However, when interpreting these findings, it is important to consider some limitations. Firstly, the diagnosis of endometriosis was based on subjects’ self-reporting, relying only on historical information, which may have limited the diagnostic accuracy. While such a definitional approach is widely used in relevant studies, it must also be noted that some subjects may have given inaccurate reports due to forgetfulness, misunderstandings, or an insufficient understanding of their disease, and future studies should adopt a more rigorous diagnostic approach. Secondly, due to the limitations of the cross-sectional study design, although we found a significant association between TyG and its obesity-related indices and EMS, we could not determine whether an increase in TyG directly leads to the occurrence of EMS. This suggests that these indicators should be interpreted cautiously in clinical applications, and future studies should integrate longitudinal data to improve the predictive power of the model. Moreover, as population trends change over time, it will be necessary for future research to conduct longitudinal studies to better understand the dynamic relationship between metabolic indicators and EMS. Furthermore, while several covariates were adjusted for, unmeasured confounding factors may still have existed, such as electrolytes, liver and renal function, muscle enzymes, sex hormones, socioeconomic status, psychological stress, and environmental pollution. These factors could have influenced the results of the analysis. Therefore, future studies should consider more comprehensive covariate adjustments and the integration of multidimensional factors to accurately elucidate the relationship between the TyG index and its derivatives and EMS, as well as the underlying mechanisms. Additionally, the classification method for covariates such as smoking and alcohol intake was not based on major international epidemiological guidelines, which could have led to overestimation of the prevalence of the related risk factors. Classifying the educational level according to the number of years of education may lead to underestimation or mask the actual impact of an individual’s educational background on the association between TyG and EMS. This suggests that future studies should incorporate multidimensional indicators to more comprehensively evaluate the moderating effect of education on the relationship between TyG and EMS. Finally, the NHANES data primarily represent the adult population in the United States. Considering the differences in genetics, lifestyles, and environmental factors across different populations, the findings of this study may not be fully generalizable to those in other countries or regions. Future research should consider cross-national comparative studies to enhance the applicability of the results.

## 5. Conclusions

This study revealed that among individuals with higher education levels, TyG and its obesity-related derivatives may be associated with a higher prevalence of EMS. This suggests that when formulating public health strategies for early intervention in EMS, greater attention should be given to TyG and its obesity-related derivatives in populations with higher educational levels.

## Figures and Tables

**Table 1:** Baseline features of LEL group versus HEL group.

	Total	LEL Group	HEL Group	*p* *
	N = 2347	N = 996	N = 1351	
Age, years	37 (28–45)	37 (28–46)	37 (28–45)	0.469
Race (n, %)				<0.001
Mexican American	484 (20.62)	312 (31.33)	172 (12.73)	
Other Hispanic	105 (4.47)	61 (6.12)	44 (3.26)	
Non-Hispanic white	1118 (47.64)	370 (37.15)	748 (55.37)	
Non-Hispanic black	535 (22.80)	218 (21.89)	317 (23.46)	
Other race	105 (4.47)	35 (3.51)	70 (5.18)	
Marital status (n, %)				0.087
Married or with partner	1509 (64.29)	660 (66.27)	849 (62.84)	
Single	838 (35.71)	336 (33.73)	502 (37.16)	
Family PIR (n, %)				<0.001
<1.3	612 (26.08)	389 (39.06)	223 (16.51)	
1.3–3.49	859 (36.60)	416 (41.77)	443 (32.79)	
≥3.5	876 (37.32)	191 (19.18)	685 (50.70)	
Drinking (n, %)				<0.001
Yes	1476 (62.89)	557 (55.92)	919 (68.02)	
No	871 (37.11)	439 (44.08)	432 (31.98)	
Smoking (n, %)				<0.001
Yes	908 (36.69)	438 (43.98)	470 (34.79)	
No	1439 (61.31)	558 (56.02)	881 (65.21)	
Hypertension (n, %)				0.053
Yes	470 (20.03)	218 (21.89)	252 (18.65)	
No	1877 (79.97)	778 (78.11%)	1099 (81.35)	
Diabetes (n, %)				0.005
Yes	112 (4.77)	62 (6.22)	50 (3.70)	
No	2235 (95.23)	934 (93.78)	1301 (96.30)	
Vigorous activity (n, %)				<0.001
Yes	850 (36.22)	227 (22.79)	623 (46.11)	
No	1497 (63.78)	769 (77.21)	728 (53.89)	
Moderate activity (n, %)				<0.001
Yes	1277 (54.41)	415 (41.67)	862 (63.80)	
No	1070 (45.59)	581 (58.33)	489 (36.20)	
Ever been pregnant (n, %)				<0.001
Yes	1957 (83.38)	922 (92.57)	1035 (76.61)	
No	390 (16.62)	74 (7.43)	316 (23.39)	
EMS (n, %)				0.024
Yes	203 (8.65)	71 (7.13)	132 (9.77)	
No	2144 (91.35)	925 (92.87)	1219 (90.23)	
WC, cm	91.20 (81.50–103.90)	93.95 (83.68–105.40)	89.20 (79.85–102.00)	<0.001
HT, cm	162.70 (158.30–167.30)	161.20 (156.70–165.90)	163.50 (159.40–168.10)	<0.001
BMI, kg/m^2^	27.24 (23.45–32.70)	28.52 (24.23–33.34)	26.62 (22.86–32.20)	<0.001
GLU, mg/dL	92.00 (86.30–99.00)	93.50 (87.57–100.93)	91.00 (85.70–97.00)	<0.001
TG, mg/dL	98 (68–144)	107 (74–155)	91 (64–133)	<0.001
Menarche age, years	12 (12–13)	12 (12–13)	12 (12–13)	0.596
TyG	8.40 (8.02–8.84)	8.52 (8.13–8.96)	8.34 (7.99–8.75)	<0.001
TyG–WHtR	4.75 (4.08–5.59)	5.04 (4.32–5.77)	4.54 (3.95–5.42)	<0.001
TyG–WC	773.58 (666.33–904.35)	809.55 (694.91–931.28)	744.89 (651.69–882.11)	<0.001
LnTyG–WC	6.65 (6.50–6.81)	6.70 (6.54–6.84)	6.61 (6.48–6.78)	<0.001
TyG–BMI	233.48 (191.40–286.03)	246.27 (202.35–293.20)	222.83 (185.57–275.97)	<0.001
LnTyG–BMI	5.45 (5.25–5.66)	5.51 (5.31–5.68)	5.41 (5.22–5.62)	<0.001

Non-parametric variables are presented as medians (interquartile range (IQR)), while categorical variables are expressed as numbers and percentages (n, %). * The Mann–Whitney U test was used to compare continuous variables with an abnormal distribution between the two groups. Categorical variables were determined using the chi-squared test. The statistical significance level was set to below 0.05. BMI = body mass index; EMS = endometriosis; HEL = high educational level; HT = height; LEL = low educational level; PIR = poverty-income ratio; TG = triglycerides; TyG = triglyceride-glucose; TyG-WC = triglyceride-glucose-waist circumference; TyG-WHtR = triglyceride-glucose-waist-to-height ratio; TyG-BMI = triglyceride-glucose-body mass index; WC = waist circumference.

**Table 2:** The relationship between TyG and its obesity-related derivatives and EMS.

	OR (95% CI), *p*								
	ALL (n = 2347)			HEL (n = 1351)			LEL (n = 996)		
	Model 1 ^1^	Model 2 ^2a^	Model 3 ^3a^	Model 1 ^1^	Model 2 ^2b^	Model 3 ^3b^	Model 1 ^1^	Model 2 ^2b^	Model 3 ^3b^
EMS									
TyG	1.53 (1.23, 1.89) <0.001	1.54 (1.22, 1.95) <0.001	1.56 (1.20, 2.02) <0.001	1.82 (1.37, 2.40) <0.001	1.73 (1.29, 2.32) <0.001	1.68 (1.21, 2.34) 0.002	1.31 (0.92, 1.88) 0.140	1.23 (0.81, 1.87) 0.335	1.253 (0.78, 2.02) 0.354
Category									
Q1	1.0	1.0	1.0	1.0	1.0	1.0	1.0	1.0	1.0
Q2	1.45 (0.93, 2.27) <0.001	1.43 (0.91, 2.24) 0.125	1.38 (0.87, 2.19) 0.166	2.73 (1.50, 4.98) 0.001	2.683 (1.46, 4.93) 0.00147	2.47 (1.32, 4.60) 0.004	1.53 (0.72, 3.25) 0.266	1.25 (0.57, 2.75) 0.573	1.10 (0.48, 2.51) 0.823
Q3	1.34 (0.86, 2.10) 0.200	1.34 (0.84, 2.12) 0.219	1.24 (0.77, 1.98) 0.374	1.89 (1.01, 3.55) 0.048	1.816 (0.96, 3.45) 0.06825	1.63 (0.85, 3.15) 0.143	1.82 (0.88, 3.79) 0.109	1.52 (0.70, 3.30) 0.293	1.41 (0.62, 3.20) 0.417
Q4	2.04 (1.34, 3.10) <0.001	2.01 (1.29, 3.13) 0.002	1.91 (1.20, 3.04) 0.006	3.23 (1.79, 5.82) <0.001	3.03 (1.65, 5.57) <0.001	2.72 (1.43, 5.15) 0.002	1.67 (0.80, 3.50) 0.171	1.24 (0.57, 2.74) 0.588	1.13 (0.48, 2.68) 0.774
*p* for trend	0.002	<0.001	0.007	<0.001	0.003	0.034	0.333	0.355	0.293
EMS									
TyG-WHtR	1.16 (1.02, 1.32) 0.028	1.20 (1.05, 1.38) 0.009	1.18 (1.01, 1.38) 0.039	1.40 (1.19, 1.65) <0.001	1.36 (1.15, 1.62) <0.001	1.30 (1.07, 1.58) 0.010	0.89 (0.71, 1.12) 0.322	0.92 (0.72, 1.17) 0.503	0.90 (0.68, 1.19) 0.445
Category									
Q1	1.0	1.0	1.0	1.0	1.0	1.0	1.0	1.0	1.0
Q2	0.97 (0.62, 1.51) 0.876	1.06 (0.67, 1.68) 0.800	1.02 (0.64, 1.63) 0.925	1.51 (0.82, 2.76) 0.183	1.47 (0.80, 2.71) 0.218	1.49 (0.80, 2.78) 0.206	0.84 (0.43, 1.61) 0.590	0.98 (0.49, 1.96) 0.960	0.80 (0.39, 1.66) 0.550
Q3	1.39 (0.92, 2.10) 0.123	1.53 (0.99, 2.36) 0.055	1.45 (0.92, 2.26) 0.107	1.96 (1.10, 3.50) 0.022	1.87 (1.03, 3.38) 0.038	1.72 (0.93, 3.16) 0.082	0.79 (0.41, 1.54) 0.484	0.98 (0.49, 1.98) 0.956	0.92 (0.43, 1.96) 0.834
Q4	1.57 (1.05, 2.36) 0.030	1.82 (1.18, 2.81) 0.007	1.64 (1.00, 2.62) 0.037	2.82 (1.62, 4.90) <0.001	2.73 (1.53, 4.86) <0.001	2.38 (1.28, 4.43) 0.006	0.69 (0.35, 1.37) 0.284	0.76 (0.37, 1.57) 0.451	0.68 (0.31, 1.53) 0.353
*p* for trend	0.008	0.002	0.015	<0.001	<0.001	0.006	0.282	0.484	0.449
EMS									
LnTyG-WC	2.44 (1.23, 4.83) 0.011	2.61 (1.27, 5.36) 0.009	2.27 (1.03, 5.03) 0.042	6.02 (2.55, 14.20) <0.001	4.95 (2.00, 12.23) <0.001	3.74 (1.36, 10.28) 0.010	0.67 (0.21, 2.16) 0.504	0.67 (0.20, 2.26) 0.520	0.54 (0.13, 2.19) 0.385
Category									
Q1	1.0	1.0	1.0	1.0	1.0	1.0	1.0	1.0	1.0
Q2	1.37 (0.87, 2.17) 0.179	1.46 (0.91, 2.33) 0.116	1.38 (0.85, 2.21) 0.190	1.24 (0.69, 2.22) 0.472	1.23 (0.68, 2.22) 0.502	1.19 (0.65, 2.17) 0.575	0.70 (0.33, 1.33) 0.304	0.77 (0.38, 1.57) 0.473	0.66 (0.31, 1.39) 0.271
Q3	1.63 (1.04, 2.55) 0.033	1.70 (1.07, 2.71) 0.024	1.62 (1.01, 2.61) 0.047	1.43 (0.81, 2.52) 0.220	1.31 (0.73, 2.36) 0.362	1.17 (0.64, 2.13) 0.618	1.05 (0.56, 1.97) 0.889	1.20 (0.61, 2.35) 0.591	1.18 (0.57, 2.43) 0.661
Q4	1.95 (1.26, 3.01) 0.003	2.13 (1.35, 3.36) 0.001	1.92 (1.18, 3.11) 0.009	2.41 (1.43, 4.07) 0.001	2.24 (1.30, 3.87) 0.004	1.89 (1.05, 3.40) 0.033	0.59 (0.29, 1.21) 0.149	0.57 (0.27, 1.23) 0.15	0.48 (0.21, 1.12) 0.089
*p* for trend	0.002	<0.001	0.007	<0.001	0.003	0.034	0.333	0.355	0.293
EMS									
LnTyG-BMI	1.60 (0.95, 2.67) 0.077	1.72 (1.00, 2.97) 0.052	1.50 (0.83, 2.72) 0.182	3.20 (1.71, 5.99) <0.001	2.78 (1.42, 5.45) 0.003	2.14 (1.01, 4.50) 0.044	0.49 (0.20, 1.21) 0.120	0.56 (0.22, 1.45) 0.233	0.45 (0.15, 1.34) 0.153
Category									
Q1	1.0	1.0	1.0	1.0	1.0	1.0	1.0	1.0	1.0
Q2	1.69 (1.02, 2.63) 0.021	1.75 (1.11, 2.75) 0.0156	1.65 (1.04, 2.62) 0.033	2.43 (1.28, 4.59) 0.006	2.28 (1.20, 4.35) 0.012	2.19 (1.14, 4.22) 0.019	0.99 (0.52, 1.91) 0.976	1.02 (0.51, 2.02) 0.960	0.84 (0.40, 1.74) 0.638
Q3	1.67 (1.07, 2.61) 0.023	1.90 (1.22, 3.06) 0.005	1.84 (1.15, 2.90) 0.012	2.68 (1.42, 5.06) 0.002	2.40 (1.25, 4.60) 0.009	2.23 (1.14, 4.35) 0.019	1.17 (0.62, 2.23) 0.625	1.34 (0.68, 2.64) 0.392	1.45 (0.70, 3.01) 0.321
Q4	1.589 (1.01, 2.49) 0.044	1.74 (1.09, 2.78) 0.021	1.50 (0.91, 2.47) 0.112	3.29 (1.77, 6.12) <0.001	3.05 (1.60, 5.80) 0.001	2.54 (1.28, 5.04) 0.008	0.47 (0.22, 1.04) 0.061	0.51 (0.23, 1.16) 0.107	0.44 (0.18, 1.06) 0.066
*p* for trend	0.079	0.030	0.141	<0.001	0.001	0.019	0.131	0.256	0.251

^1^ Model 1: This model did not include any adjustments. ^2a^ Model 2: Adjustments were made for age, race, marital status, education level, and family PIR. ^2b^ Model 2: Adjusted for age, race, marital status, and family PIR. ^3a^ Model 3: Further adjustments based on 2a Model 2 included drinking status, smoking status, hypertension, diabetes, vigorous activity, moderate activity, menarche age, and pregnancy history. ^3b^ Model 3: Further adjustments based on 2b Model 2 included drinking status, smoking status, hypertension, diabetes, vigorous activity, moderate activity, menarche age, and pregnancy history. CI = confidence interval; EMS = endometriosis; HEL = high educational level; LEL = low educational level; OR = odds ratio; TyG = triglyceride-glucose; TyG-WC = triglyceride-glucose-waist circumference; TyG-WHtR = triglyceride-glucose-waist-to-height ratio; TyG-BMI = triglyceride-glucose-body mass index.

**Table 3:** Threshold effect analysis of TyG and its obesity-related derivatives in HEL group.

EMS	TyG	TyG-WHtR	LnTyG-WC	LnTyG-BMI
	OR (95% CI), *p*			
Linear regression model	1.718 (1.231, 2.397) 0.0015	1.297 (1.065, 1.580) 0.0098	3.759 (1.359, 10.397) 0.0107	2.127 (1.008, 4.488) 0.0477
Two-segment piecewise linear regression model				
Inflection point	7.99	6.03	6.92	5.92
Index < inflection point	12.065 (1.300, 111.974) 0.0285	1.556 (1.192, 2.031) 0.0011	6.327 (1.863, 21.490) 0.0031	3.461 (1.490, 8.043) 0.0039
Index > inflection point	1.418 (0.960, 2.093) 0.0790	0.684 (0.346, 1.353) 0.2754	0.067 (0.000, 14.904) 0.3273	0.000 (0.000, 1.241) 0.0562
Log-likelihood ratio	0.051	0.041	0.119	0.009

CI = confidence interval; EMS = endometriosis; HEL = high educational level; OR = odds ratio; TyG = triglyceride-glucose; TyG-WC = triglyceride-glucose-waist circumference; TyG-WHtR = triglyceride-glucose-waist-to-height ratio; TyG-BMI = triglyceride-glucose-body mass index.

## Data Availability

Data are contained within the article.

## Supplementary Materials

The following supporting information can be downloaded at https://www.mdpi.com/article/10.3390/nu17040670/s1. Table S1: Univariable logistic regression analysis of TyG and its obesity-related derivatives and EMS. Table S2: Multivariable logistic regression analysis of TyG and its obesity-related derivatives and EMS. Figure S1. Schematic diagram of the association between TyG and TyG-related index with endometriosis varies by educational level.

## Abbreviations

TyG = triglyceride-glucose; EMS = endometriosis; TyG-WC = triglyceride-glucose-waist circumference; TyG-WHtR = triglyceride-glucose-waist-to-height ratio; TyG-BMI = triglyceride-glucose-body mass index; NHANES = National Health and Nutrition Examination Survey; TGs = triglycerides; FG = fasting glucose; LEL = low educational level; HEL = high educational level; PIR = poverty-income ratio; OR = odds ratio; CI = confidence interval.
