# Supplementary material for: Association Between Triglyceride–Glucose Related Index and Endometriosis Varies According to Educational Level
Source: Nutrients. 2025 Feb 13;17(4):670. doi: 10.3390/nu17040670 (PMC11858264; doi:10.3390/nu17040670)
Supplement: Supplementary file 1 [file nutrients-17-00670-s001.zip › nutrients-3426406-supplementary.pdf]

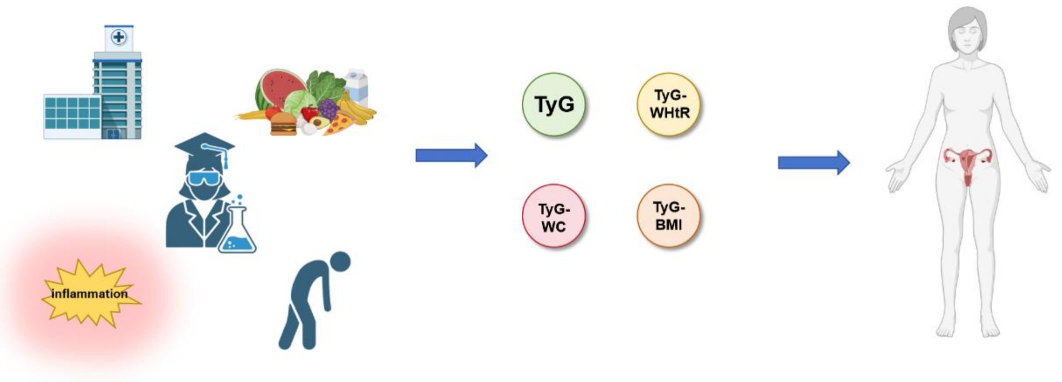

Figure S1. Schematic diagram of the association between TyG and TyG-related index with endometriosis varies by educational level.

Table S1. Univariable logistic regression analysis of TyG and Its Obesity-Related Derivatives with EMS.

	**ALL (n=2347)**		**HEL (n=1351)**		**LEL (n=996)**	
	**OR (95% CI)**	***P***	**OR (95% CI)**	***P***	**OR (95% CI)**	***P***
**Age, years**	1.046 (1.031, 1.062)	<0.001	1.055 (1.035, 1.076)	<0.001	1.033 (1.008, 1.058)	0.010
**Race (n, %)**						
Mexican American	1.0		1.0		1.0	
Other Hispanic	4.353 (1.638, 11.564)	0.003	2.767 (0.745, 10.268)	0.128	7.228 (1.576, 33.161)	0.011
Non-Hispanic White	7.493 (3.785, 14.835)	<0.001	3.453 (1.482, 8.046)	0.004	18.369 (5.689, 59.309)	<0.001
Non-Hispanic Black	4.380 (2.106, 9.111)	<0.001	3.434 (1.414, 8.337)	0.006	2.915 (0.721, 11.785)	0.133
Other Race	3.199 (1.113, 9.190)	0.031	1.677 (0.458, 6.134)	0.435	6.242 (1.007, 38.715)	0.049
**Education level(n, %)**						
LEL	1.0					
HEL	1.411 (1.044, 1.906)	0.025				
**Marital status (n, %)**						
Married or with partner	1.0		1.0		1.0	
Single	0.877 (0.646, 1.191)	0.401	0.862 (0.591, 1.259)	0.443	0.874 (0.519, 1.471)	0.611
**Family PIR (n, %)**	1.192 (1.090, 1.303)	<0.001	1.139 (1.012, 1.282)	0.031	1.234 (1.055, 1.444)	0.008
**Drinking (n, %)**						
Yes	1.0		1.0		1.0	
No	0.744 (0.546, 1.014)	0.062	1.112 (0.761, 1.625)	0.583	0.407 (0.235, 0.705)	0.001
**Smoking` (n, %)**						
Yes	1.0		1.0		1.0	
No	0.559 (0.419, 0.746)	<0.001	0.698 (0.485, 1.005)	0.053	0.326 (0.194, 0.548)	<0.001
**Hypertension (n, %)**						
Yes	1.0		1.0		1.0	
No	0.628 (0.453, 0.870)	0.005	0.574 (0.381, 0.866)	0.008	0.694 (0.405, 1.192)	0.186
**Diabetes (n, %)**						
Yes	1.0		1.0		1.0	
No	1.088 (0.542, 2.184)	0.813	0.653 (0.288, 1.482)	0.308	2.393 (0.573, 10.000)	0.232
**Vigorous activity (n, %)**						
Yes	1.0		1.0		1.0	
No	0.881 (0.656, 1.185)	0.403	1.140 (0.794, 1.639)	0.477	0.634 (0.375, 1.074)	0.090
**Moderate activity (n, %)**						
Yes	1.0		1.0		1.0	
No	1.010 (0.756, 1.348)	0.947	1.292 (0.897, 1.862)	0.169	0.810 (0.499, 1.315)	0.394
**Ever been pregnant (n, %)**						
Yes	1.0		1.0		1.0	
No	0.972 (0.658, 1.435)	0.885	1.054 (0.692, 1.603)	0.808	0.167 (0.023, 1.216)	0.077
**BMI, kg/m2**	1.007 (0.988, 1.026)	0.474	1.030 (1.007, 1.052)	0.009	0.960 (0.925, 0.997)	0.033
**Menarche age, years**	0.942 (0.864, 1.026)	0.173	0.891 (0.800, 0.992)	0.036	1.044 (0.904, 1.205)	0.558
**TyG**	1.525 (1.229, 1.893)	<0.001	1.817 (1.374, 2.402) 0.00003	<0.001	1.311 (0.915, 1.879)	0.140
**TyG-WHtR**	1.158 (1.016, 1.319)	0.028	1.403 (1.193, 1.650)	<0.001	0.889 (0.705, 1.121)	0.322
**LnTyG-WC**	2.435 (1.228, 4.829)	0.011	6.016 (2.550, 14.193)	<0.001	0.670 (0.208, 2.164)	0.504
**LnTyG-BMI**	1.595 (0.951, 2.674)	0.077	3.196 (1.705, 5.991)	<0.001	0.486 (0.196, 1.205)	0.119

BMI, body mass index; EMS, endometriosis; HEL,high educational level; LEL, low educational level; NA, not available;PIR,poverty-income ratio;TyG,triglyceride-glucose; TyG-WC, triglyceride-glucose waist circumferen-

ce; TyG-WHtR, triglyceride-glucose waist-to-height ratio; TyG-BMI, triglyceride-glucose body mass index.

Table S2. Multivariable logistic regression analysis of TyG and Its Obesity-Related Derivatives with EMS.

	ALL (n=2347)		HEL (n=1351)		LEL (n=996)	
	**OR (95% CI)**	***P***	**OR (95% CI)**	***P***	**OR (95% CI)**	***P***
**Age, years**	1.03 (1.02, 1.05)	<0.001	1.04 (1.02, 1.07)	<0.001	1.02 (1.00, 1.05)	0.097
**Race (n, %)**						
Mexican American	1.0		1.0		1.0	
Other Hispanic	4.67 (1.74, 12.50)	0.002	3.45 (0.91, 13.04)	0.068	6.91 (1.49, 32.02) 0.01354	0.014
Non-Hispanic White	6.28 (3.12, 12.66)	<0.001	3.27 (1.39, 7.72)			
Non-Hispanic Black		0.007	13.78 (4.14, 45.56)	<0.001		
Other Race	4.44 (2.10, 9.40)	<0.001	3.40 (1.37, 8.43)			
**Education level(n, %)**		0.008	2.85 (0.69, 11.70)	0.146		
LEL	2.90 (1.00, 8.44)	0.051	1.80 (0.44, 6.70)			
HEL		0.381	5.36 (0.85, 33.61)	0.073		
**Marital status (n, %)**						
Married or with partner	1.0		NA		NA	
Single	1.21 (0.87, 1.70)	0.258	NA		NA	
**Family PIR (n, %)**						
**Drinking (n, %)**	NA		NA		NA	
Yes	NA		NA		NA	
No	1.07 (0.97, 1.19) 0.17518	0.175	1.08 (0.95, 1.23)	0.226	1.03 (0.87, 1.22)	0.716
**Smoking` (n, %)**						
Yes	NA		NA		1.0	
No	NA		NA		0.64 (0.35, 1.17)	0.148
**Hypertension (n, %)**						
Yes	1.0				1.0	
No	0.71 (0.52, 0.97)	0.029			0.64 (0.35, 1.17)	0.141
**Diabetes (n, %)**						
Yes	1.0		1.0		NA	
No	0.85 (0.59, 1.21)	0.356	0.85 (0.54, 1.32)	0.47	NA	NA
**Vigorous activity (n, %)**						
Yes	NA		NA		NA	
No	NA		NA		NA	NA
**Moderate activity (n, %)**						
Yes	NA		NA		NA	
No	NA		NA		NA	NA
**Ever been pregnant (n, %)**						
Yes	NA		NA		NA	
No	NA		NA		NA	NA
**BMI, kg/m2**						
**Menarche age, years**	NA		NA		NA	
**TyG**	NA		NA		NA	NA
**TyG-WHtR**	NA		0.89 (0.80, 1.99)	0.045	NA	NA
**LnTyG-WC**	1.48 (1.16, 1.21)	0.002	1.63 (1.21, 2.21)	0.002	1.21 (0.79, 1.85)	0.389
**LnTyG-BMI**	1.17 (1.01, 1.35)	0.032	1.29 (1.08, 1.56)	0.006	0.92 (0.72, 1.18)	0.516

BMI, body mass index; EMS, endometriosis; HEL,high educational level; LEL, low educational level; NA, not available;PIR,poverty-income ratio;TyG,triglyceride-glucose; TyG-WC, triglyceride-glucose waist circumference; TyG-WHtR, triglyceride-glucose waist-to-height ratio; TyG-BMI, triglyceride-glucose body mass index.
